# Adaptation to compound climate risks: A systematic global stocktake

**DOI:** 10.1016/j.isci.2023.105926

**Published:** 2023-01-04

**Authors:** Nicholas P. Simpson, Portia Adade Williams, Katharine J. Mach, Lea Berrang-Ford, Robbert Biesbroek, Marjolijn Haasnoot, Alcade C. Segnon, Donovan Campbell, Justice Issah Musah-Surugu, Elphin Tom Joe, Abraham Marshall Nunbogu, Salma Sabour, Andreas L.S. Meyer, Talbot M. Andrews, Chandni Singh, A.R. Siders, Judy Lawrence, Maarten van Aalst, Christopher H. Trisos, The Global Adaptation Mapping Initiative Team

**Affiliations:** 1 African Climate and Development Initiative, University of Cape Town, Cape Town, South Africa; 2 CSIR-Science and Technology Policy Research Institute, Accra, Ghana; 3 Department of Environmental Science and Policy, Rosenstiel School of Marine, Atmospheric, and Earth Science, and Leonard and Jayne Abess Center for Ecosystem Science and Policy, University of Miami, Miami, FL, USA; 4 Priestley International Centre for Climate, University of Leeds, Leeds, UK; 5 Wageningen University, Wageningen, the Netherlands; 6 Deltares, Delft, the Netherlands, Department of Physical Geography, Utrecht University, Utrecht, the Netherlands; 7 Alliance of Bioversity International and International Center for Tropical Agriculture (CIAT), Dakar, Senegal, Faculty of Agronomic Sciences, University of Abomey-Calavi, Cotonou, Benin; 8 The University of West Indies, Mona, Jamaica; 9 United Nations University, Bonn, Germany; 10 Department of Public Administration and Health Service Management, University of Ghana, Legon, Ghana; 11 Economics Center, World Resources Institute, New Delhi, India; 12 Department of Geography and Environmental Management, University of Waterloo, Waterloo, ON, Canada; 13 Faculty of Engineering and Physical Sciences, University of Southampton, Highfield, Southampton, UK; 14 Department of Political Science, University of Connecticut, Storrs, CT, USA; 15 School of Environment and Sustainability, Indian Institute for Human Settlements, Bangalore, India; 16 Disaster Research Center, Climate Change Science and Policy Hub, Biden School of Public Policy, Department of Geography and Spatial Sciences; University of Delaware; Newark, DE, USA; 17 Climate Change Research Institute, Victoria University of Wellington, Wellington, New Zealand; 18 Faculty of Geo-information Science and Earth Observation, University of Twente, Twente, the Netherlands; 19 Red Cross Red Crescent Climate Centre, The Hague, The Netherlands; 20 Centre for Statistics in Ecology, Environment and Conservation, University of Cape Town, Cape Town, South Africa

**Keywords:** Earth sciences, Climatology, Safety engineering, Business, Decision science

## Abstract

This article provides a stocktake of the adaptation literature between 2013 and 2019 to better understand how adaptation responses affect risk under the particularly challenging conditions of compound climate events. Across 39 countries, 45 response types to compound hazards display anticipatory (9%), reactive (33%), and maladaptive (41%) characteristics, as well as hard (18%) and soft (68%) limits to adaptation. Low income, food insecurity, and access to institutional resources and finance are the most prominent of 23 vulnerabilities observed to negatively affect responses. Risk for food security, health, livelihoods, and economic outputs are commonly associated risks driving responses. Narrow geographical and sectoral foci of the literature highlight important conceptual, sectoral, and geographic areas for future research to better understand the way responses shape risk. When responses are integrated within climate risk assessment and management, there is greater potential to advance the urgency of response and safeguards for the most vulnerable.

## Highlights

• Compound climate impacts are particularly hard to adapt to
• Compound vulnerabilities and exposures constrain adaptation capabilities
• Inappropriate responses to climate change can lead to maladaptation
• Compound impacts can have cascading effects on response options

## Graphical abstract

**Figure:**
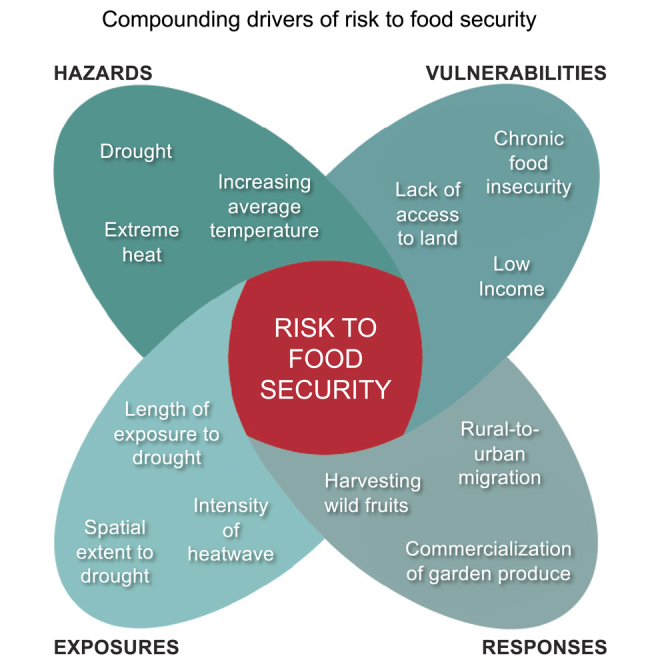

## Introduction

Urgent and widespread responses to climate change are needed within the next decade to minimize loss and damage from climate change and keep global warming under the risk threshold of 2°C and, if possible, under 1.5°C.^1^^,^^2^^,^^3^^,^^4^ Current progress toward these goals remains insufficient for both climate change mitigation and adaptation.^4^^,^^5^^,^^6^^,^^7^ There is limited evidence that observed adaptation-related responses are reducing risks posed by climate change,^8^^,^^9^^,^^10^^,^^11^ and there is growing concern that inappropriate responses are increasing vulnerability and leading to maladaptation.^12^^,^^13^^,^^14^ This problem is only amplified by impacts from compound climate events, the outcomes of the combination of multiple drivers and/or hazards that contribute to societal or environmental risk.^15^^,^^16^^,^^17^

Human-induced climate change is already affecting weather and climate extremes, such as heat waves, heavy precipitation, droughts, and tropical cyclones, in every region across the globe.^15^^,^^18^ For example, temperature extremes (including heat waves) have become more frequent and more intense across most land regions since the 1950s.^15^ Importantly, human-induced climate change has also increased the chance of compound extreme impacts, such as observed extreme rainfall following wildfires in the western United States.^19^^,^^20^ Furthermore, compound interactions between multiple extremes such as extreme marine heat waves and extreme ocean acidity can have larger impacts on marine ecosystems than the individual extremes.^21^ There is also increased severity of risk associated with these events due to amplifications in their magnitude, spatial extent, or frequency. For example, the effects of sea level rise combined with increases in storm surge frequency or magnitude amplify the likelihood of extreme impacts,^22^ or a storm surge in combination with extreme rainfall,^23^ or a combination of hot, dry, and windy conditions that create fire weather conditions.^15^^,^^24^ Many regions are projected to experience an increase in the probability of compound events with higher global warming.^15^ These are also compounded by increasing exposure and vulnerability to compound climate impacts across the world.^25^^,^^26^^,^^27^^,^^28^

Risks from climate change differ through space and time and cascade across and within regions and systems.^16^^,^^29^ Research on compound climate events has made significant progress in recent years, quantifying compound relationships and risk associated with extreme events,^15^^,^^30^^,^^31^ including those attributed to human-caused climate change.^32^^,^^33^ Yet, less is known about how responses to compound climate events affect each other and overall risk.^18^^,^^34^^,^^35^^,^^36^ Furthermore, we lack an empirical assessment of how social and political determinants of risk interact and affect the multiple determinants of exposure, response, and vulnerability.^29^ This is particularly important for adaptation as the greatest near-term gains in risk reduction can be made through enhancing responses that reduce exposures and vulnerabilities across vulnerable communities and the Global South.^9^^,^^18^ Yet, effective risk management, as well as assessment of the feasibility of our responses to climate change, requires knowledge of how social, political, technical, and environmental drivers interact to shape compound risks.^10^^,^^37^ We need to match our emerging understanding of risk with how best to respond to climate change.

In order to fill this gap, we use a systematic global stocktake of human responses to climate change published between 2013 and 2019, the Global Adaptation Mapping Initiative (GAMI).^8^^,^^38^ The global adaptation literature is a useful lens to gain a better understanding of the response interactions that affect risk because by definition “adaptation” is a form of climate risk reduction.^9^^,^^35^ However, congruent with the Intergovernmental Panel on Climate Change (IPCC) usage, we use the term “adaptation-related responses”, recognizing that not all responses reduce risk. While “adaptation” implies risk reduction, we use the broader term “responses” to reflect that responses may decrease risk or may, in some cases, increase risk and lead to maladaptation.^9^ Climate responses may face limits,^39^ fail to achieve their objectives, involve trade-offs among objectives or across stakeholders, involve unintended consequences for other groups or societal objectives, or increase risk to other climate risk drivers.^40^ These challenges are particularly stark in response to compound events.^35^ Just as we use the term “response” to encompass a wide range of actions, we use a holistic definition of “risk” that incorporates hazards, exposure, vulnerability, and response.^29^

Focusing on human responses to compound climate impacts is also particularly important given the current slow pace of climate action and the need to rapidly scale up both adaptation and mitigation responses to climate change over the next decade.^3^^,^^4^^,^^8^^,^^9^ Furthermore, we urgently need knowledge on the feasibility of adaptation options,^10^^,^^37^ particularly for ensuring responses to climate change do not compromise other response options and increase vulnerability, exposure, or overall risk.^29^

### The challenges of adapting to compound climate impacts

Changing climate-driven impacts can compound over different dimensions of time and space as well as with other determinants of climate change risk.^15^^,^^16^^,^^29^^,^^30^ Climate impacts can interact with the level of exposure and vulnerability of societal systems to climate change as well as direct and indirect responses to climate change.^41^^,^^42^ That is, climate-related hazards can interact with vulnerability, exacerbating the severity of the event (see Table 1 for explanation of risk terminologies used in this article). For example, changes in heat, precipitation, evapotranspiration, and soil moisture can lead to the compound extreme of drought and extreme heat,^30^ but the societal influence on the severity of the risk can be affected by manifold societal drivers of risk such as poverty, gender, water management, and land-use change.^43^^,^^44^

**Table 1:** Risk terminologies used in this article

Term	Definition	Reference
Adaptation	The process of adjustment to actual or expected climate and its effects, in order to moderate harm or exploit beneficial opportunities	O'Neill et al., 2022, IPCC, 2022, Ara Begum et al., 2022^9^^,^^45^^,^^46^
Cascading impacts	Cascading impacts from extreme weather/climate events occur when an extreme hazard generates a sequence of secondary events in natural and human systems that result in physical, natural, social, or economic disruption, whereby the resulting impact is significantly larger than the initial impact. Cascading impacts are complex and multidimensional and are associated more with the magnitude of vulnerability than with that of the hazard.	IPCC, 2022, Pescaroli and Alexander, 2015, Pescaroli and Alexander, 2018^45^^,^^47^^,^^48^
Climate change risk	The potential for adverse consequences for human or ecological systems, recognizing the diversity of values and objectives associated with such systems. In the context of climate change, risks can arise from potential impacts of climate change as well as human responses to climate change. Hazards, exposure, and vulnerability may each be subject to uncertainty in terms of magnitude and likelihood of occurrence, and each may change over time and space due to socio-economic changes and human decision-making.	IPCC, 2022^45^
Compound weather/climate event	The terms “compound events”, “compound extremes”, and “compound extreme events” are used interchangeably in the literature and this report and refer to the combination of multiple drivers and/or hazards that contributes to societal and/or environmental risk.	IPCC, 2021, Zscheischler et al., 2018^15^^,^^16^
Compound climate impact	The risk outcome from the combination of multiple drivers and/or hazards that contributes to societal and/or environmental risk. For example, heat and moisture extremes and their impacts become compounded through crop-physiological interactions, heat-moisture couplings in the climate system, and crop-atmosphere interactions.	Lesk et al., 2022^49^
Compound climate risks	Arise from the interaction of climate change risks, which may be characterized by single extreme events or multiple coincident or sequential events that interact with exposed systems or sectors. For example, a multi-breadbasket failure can affect financial, food, and human security through major financial losses to agricultural insurers globally and enhanced potential for civil unrest.	Simpson, et al., 2021^29^
Exposure	The presence of people; livelihoods; species or ecosystems; environmental functions, services, and resources; infrastructure; or economic, social, or cultural assets in places and settings that could be adversely affected.	IPCC, 2022, Ara Begum et al., 2022^45^^,^^46^
Hard limit to adaptation	The point at which an actor’s objectives (or system needs) cannot be secured from intolerable risks through adaptive actions. Hard adaptation limit – No adaptive actions are possible to avoid intolerable risks.	O'Neill et al., 2022, IPCC, 2022^9^^,^^45^
Impact	The consequences of realized risks on natural and human systems, where risks result from the interactions of climate-related hazards (including extreme weather/climate events), exposure, vulnerability, and responses. Impacts generally refer to effects on lives, livelihoods, health and well-being, ecosystems and species, economic, social, and cultural assets, services (including ecosystem services), and infrastructure. Impacts may be referred to as consequences or outcomes and can be adverse or beneficial.	IPCC, 2022^45^
Response	Risks result from the potential for such responses not achieving the intended objective(s) or from potential trade-offs with, or negative side effects on, other societal objectives, such as the Sustainable Development Goals (SDGs). Risks can arise, for example, from uncertainty in the implementation, effectiveness or outcomes of climate policy, climate-related investments, technology development or adoption, and system transitions.	O'Neill et al., 2022, IPCC, 2022, Ara Begum et al., 2022^9^^,^^45^^,^^46^
Soft limit to adaptation	The point at which an actor’s objectives (or system needs) cannot be secured from intolerable risks through adaptive actions. Soft adaptation limit – Options may exist but are currently not available to avoid intolerable risks through adaptive action.	O'Neill et al., 2022, IPCC, 2022^9^^,^^45^
Vulnerability	The propensity or predisposition to be adversely affected. Vulnerability encompasses a variety of concepts and elements, including sensitivity or susceptibility to harm and lack of capacity to cope and adapt.	IPCC, 2022, Ara Begum et al., 2022^45^^,^^46^

In addition to exposure and vulnerability, impacts may also interact with actions in response to climate change itself, both adaptation and mitigation.^3^^,^^29^^,^^36^^,^^50^ For example, one response to greenhouse gas emissions (GHG) mitigation is afforestation. However, when implemented inappropriately, the carbon sequestration potential of afforestation has trade-offs with other critical needs such as food security, groundwater, and soil fertility, presenting risks to biodiversity and other developmental goals.^51^ Challenges of interactions between responses also occur between adaptation options and broader development and sustainability outcomes.^18^^,^^40^ For example, this can be achieved through identifying opportunities to increase agricultural productivity over currently water-limited rain-fed croplands by adopting irrigation practices that do not deplete freshwater stocks and impair aquatic ecosystems. Expanding sustainable irrigation may avert agricultural expansion but can create additional externalities including potentially negative impacts on food security, hydroclimatic conditions, water quality, soil salinization, water storage infrastructure, and energy use that are often neglected.^52^^,^^53^^,^^54^ Increases in fire weather can also limit mitigation potential of forestry.^55^ Adaptation and mitigation efforts may also have inadvertent adverse consequences for other social goals, as when efforts to design increasingly “walkable” cities may limit accessibility for people with limited mobility due to age or ability.^56^

Impacts can also compound spatially when multiple connected locations are affected by the same or different hazards within a limited time window, thereby causing a more severe impact.^31^ Spatial compounding of climate extremes and slow emerging climate change impacts like sea level rise and responses to climate change are important for understanding emerging transboundary risks such as multiple breadbasket failures.^29^^,^^57^ Responses to spatially compounding events require capabilities that go beyond a single site of impact or group of actors to include transboundary solutions to climate risk as well as capability to manage cascading effects across multiple sectors.^18^^,^^29^^,^^58^ As urban supply chains cover increasingly large geographic regions, the potential for spatially compound hazards also increases (e.g., consider risks to ports and implications for global shipping^59^).

Impacts can also compound temporally, when a succession of hazards affect a given geographical region, leading to or amplifying an impact when compared with a single hazard (also sometimes called a cascading hazard).^31^^,^^60^ Extreme coastal storms can have temporally compounding consequences resulting in ongoing shoreline retreat and inundation projected to result from sea level rise for centuries.^61^ The impacts from such events overlap both spatially and temporally, while recovery is still underway.^3^^,^^62^ Successive impacts from extreme events place a large burden on response options, reducing response capabilities due to investment in the response to a prior event.^63^ The global COVID-19 pandemic, for example, saw many places struggle to invest in both pandemic response and hazard mitigation or recovery simultaneously. The pandemic also raised additional challenges such as safety in evacuation centers.^64^ Similarly some climate adaptation responses may reduce capacity of actors, at different scales, to engage in future responses. For example, when people leave highly hazardous areas, through displacement, migration, or managed relocation, the community that remains may have a reduced tax base or reduced social capital to implement future risk mitigation efforts.^65^^,^^66^

It is increasingly recognized that addressing multiple social drivers of climate risk equitably is essential for addressing compound climate impacts. These include significant investments in institutions for improved risk management governance and high integrated management such as improved early warning and emergency response to complement structural measures such as levees.^9^^,^^67^^,^^68^ These also include investments in infrastructure, economies, and social systems that shape how people experience and respond to exposure to climate hazards, such as housing quality, land tenure, and access to credit and insurance. Implemented measures for managing risk and curbing vulnerability following one event might not make a society sufficiently adaptive to reduce the risk from unprecedented subsequent events, which are increasing in a changing climate.^67^^,^^69^ Recent analysis has shown adaptation to compound climate events is nearly always limited and that the impact of a second event is not reduced by risk-management strategies not only for unprecedented flood or drought events of a magnitude not previously experienced^67^ but also in cases when the second event is less extreme than the first one.^67^^,^^69^

These compound effects, and their amplification of risk, present greater challenges to the risk-reduction outcomes of human responses to climate change. We currently do not know if existing or future adaptation options, including technologies, behaviors, and practices over space and time, are going to be sufficient to safeguard human and environmental needs at current and future global warming levels.^8^^,^^35^^,^^39^ We also have little understanding of how responses to multiple climate risks, and particularly compound climate events, interact and affect other adaptation and mitigation responses, nor overall risk.^29^ The governance of compounding climate risks has been highlighted as a critical enabler in this context.^68^

It is with these climatic and response imperatives that we set out to explore what we know about implemented adaptation and how best to respond to compound climate impacts.

## Results

### Hazards

**Figure 1:**
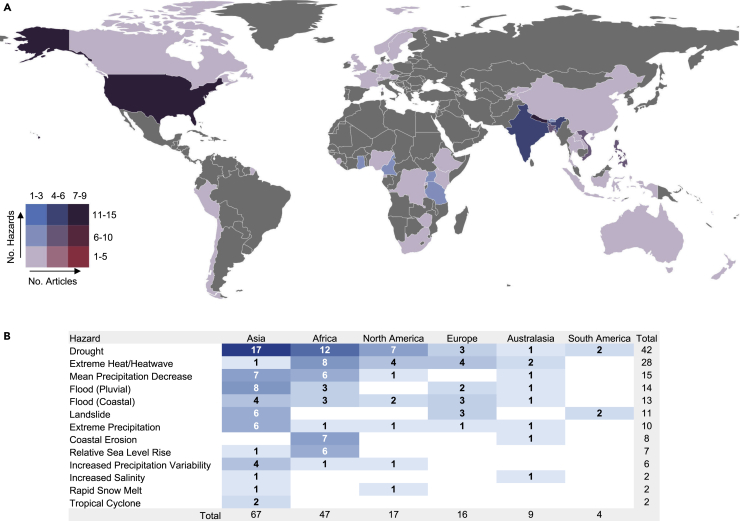
Global distribution of literature on adaptation-related responses to compound climate impacts (A and B) Bivariate map (A) and regional table (B) showing the number of papers (n = 53) and the number of compounding hazards (n = 160) per country and region of focus for the 15 most commonly reported compounding hazards in the adaptation literature on human responses to compound climate events (2013-2019).

The 53 studies we analyzed spanned 39 countries with over 45 documented types of adaptation-related responses to 24 types of compound climate events (Figure 1). While the United States dominates the focus of studies, the Americas have relatively few studies, particularly across South and Central America where there are a total of two studies: one study in Peru and one in Chile. There is a dearth of studies from eastern and southern Europe. Likewise, the Middle East, Russia, and North Africa are not well represented in the literature. However, east and central Africa are relatively well represented with multiple studies from Kenya, Uganda, and Tanzania, while Australasia is not well represented. The countries in West Africa and southern Africa with research on compound climate events align strongly with those countries that have received greater attention from climate change research funders, such as Ghana and South Africa.^70^ Across Asia, countries that have seen the most research attention on compound hazards include Nepal, India, Vietnam, Bangladesh, Bhutan, and the Philippines. This geographic distribution in the adaptation literature does not represent the global distribution of compound climate events that have increased in frequency, intensity, and extent in every region of the world.^15^

More than half (56%) of the adaptation literature describes the compound interaction between hazards qualitatively. However, quantitative description is provided for 23% of articles that show temporally compounding interactions and 15% that show spatially compounding interactions. Using the literature’s identification of qualitatively and quantitatively compounding events, the highest number of compounding hazards in the adaptation literature is associated with changes in rainfall. Notable climate hazards that compound with drought include extreme heat across the USA, Philippines, Nepal, Zimbabwe, and South Africa^71^^,^^72^^,^^73^^,^^74^^,^^75^; mean precipitation decrease in Sierra Leone, Ghana, and Bangladesh;^76^^,^^77^^,^^78^ pluvial flooding across Nepal, Uganda, Lao, and Vietnam;^79^^,^^80^^,^^81^^,^^82^ and wildfire in the USA.^83^ While 20 (38%) articles did describe the responses to spatially or temporally compound impacts or events, 33 (62%) articles did not refer explicitly to compound climate events and therefore did not fully acknowledge the compound nature of the climate events associated with the observed impacts. Nonetheless, the focus on responses to the compound climate impacts did elicit understanding of the extreme nature of the impact of such events, even if only qualitatively described.

Climate hazards that commonly compound with river flooding in the adaptation literature include extreme heat in Ghana,^84^ extreme precipitation in Sweden,^85^ storm surge in Vietnam,^86^ drought in Kenya and Vanuatu,^87^^,^^88^ and coastal flooding in the Philippines.^89^ Extreme precipitation is also observed to compound with drought across Uganda, Indonesia, and the USA^90^^,^^91^^,^^92^ and snowstorm in Tibet.^93^ Increased precipitation variability also compounds with landslides in India^94^ and windstorms in the Philippines.^95^

Across compounding hazards at the coast, sea level rise is observed to compound with coastal erosion in Cameroon and with increased salinity in Vietnam.^96^^,^^97^ Coastal erosion compounds with increased salinity in New Zealand.^98^ Coastal flooding compounds with mean precipitation decrease in Tanzania,^99^^,^^100^ with drought in Bangladesh,^101^ and with river flooding in Indonesia.^102^ Tropical cyclones compound with extreme heat in Bangladesh^103^ and extreme precipitation in India.^104^

Temporally compounding hazard interactions may be particularly challenging for adaptation when there are multiple timescales involved. For example, hydrological drought, extreme heat, and excessive evapotranspiration all act at different timescales, but they may interact to create additional challenges for response. As was the case in the case of a Great Plains drought that forced over 80 unprecedented water conservation measures in McCook, Nebraska, USA.^71^ Long-term drought and dry soils contributed to the unprecedented 2020 wildfires in Brazil.^105^ In this case, the vulnerability in the northern forested areas was higher than in the other areas, revealing a synergistic effect and temporal sequencing between fuel availability and weather-hydrological conditions.^105^ Similarly, the interactions between different types of flooding in Northern Europe (e.g., coastal and fluvial), each with their own return period, make it challenging to calculate the overall likelihood of compound floods and therefore make it difficult to evaluate appropriate risk-reduction measures.^106^

Spatially compounding interactions present their own challenges to adaptation. For example, the interaction of agricultural drought and pluvial flooding over large areas in Laos and the Philippines spatially compounded with each other and with climatic preconditions of increasing average temperature and evapotranspiration that had particularly severe impacts on livelihoods and food security of small-scale rice farmers.^81^ Adaptation across scales may be further complicated by jurisdictional and sectoral boundaries for the adaptation actors involved (i.e., the actor responsible for local flood response may not be the same actor responsible for regional drought).

### Exposures

Exposure is a physical condition resulting from being in dangerous proximity to a climate hazard without adequate protection and can be affected by the duration, intensity, and spatial extent of the event. The primary exposure characteristics affecting responses to compound climate events are relatively evenly split between duration of exposure to climate hazard (34%), spatial extent of exposure to climate hazard (32%), and intensity/magnitude of climate hazard (26%).

Length time of exposure to climate hazard is well demonstrated in three rice-growing areas of Pemba Island, Tanzania, where rice farmers were highly exposed to sea level rise and coastal floods, whose severity will increase over time as sea levels rise.^99^ The accumulating harms from repeated coastal floods and repeated losses to livelihood and productivity lead to increased dependency on imported food.^99^

Spatial extent of exposure to climate hazard has a significant effect on responses to compound climate events. For example, pastoralists whose livelihoods cover a large mobility range extent have been noted to experience risk to food security and risk of loss of livelihoods under drought conditions that restricted mobility.^80^^,^^93^ Furthermore, poor mangrove cover and the lowland elevation are the main factors explaining high exposure to sea level rise and coastal erosion for communities of the Kribi-Campo coastal ecosystems, South Cameroon.^107^ By their nature, compound climate events are associated with intensity or magnitude of climate hazards regardless of their temporal or spatial characteristics.^108^^,^^109^ One example which demonstrates the effect of the intensity of impact from the level of exposure is shown in the increased average day and night temperature of the heat wave that affected multiethnic and low-income urban communities in Western Sydney, Australia.^109^

The adaptation literature does not commonly describe the interactions between multiple exposures. When they do, interactions between the primary and secondary exposure are more commonly qualitatively described with reference to either spatial or temporal dimensions rather than both time and space (55% of articles), while quantitative descriptions of spatially compounding exposures are provided in 15% of articles and temporally compounding exposures in 11% of articles. It is also common for the adaptation literature to reference the intensity of the hazard rather than being specific on time or space dimensions of exposure (26% of articles).

### Vulnerabilities

**Figure 2:**
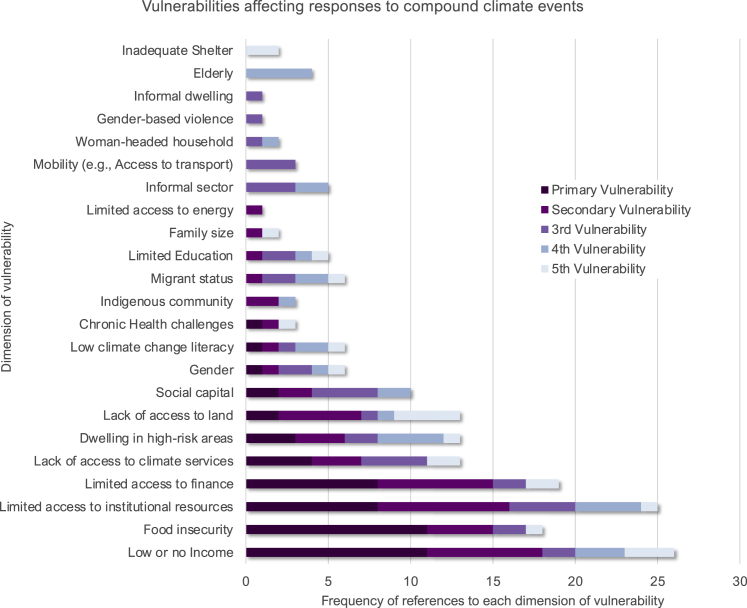
Vulnerabilities affecting responses to compound climate events Across all adaptation-related responses to compound climate events, analysis of articles identified the most important vulnerability affecting response (primary vulnerability) followed by other dimensions of vulnerability affecting response reported (secondary, third, fourth, and fifth vulnerability).

Across the adaptation literature, the most common vulnerabilities that affect responses to compound climate impacts include low or no income, food insecurity, limited access to institutional resources, and limited access to finance. However, there are at least 23 dimensions of vulnerability which affect responses under compound climate events, highlighting the diversity of contexts and multiplicity of aspects affecting vulnerability to climate change (Figure 2). The frequency of recorded dimensions of vulnerability that affect responses to compound climate extremes indicates that there are some vulnerabilities more commonly identified in the adaptation literature (e.g., low income and food insecurity) than others (e.g., shelter and elderly or gender-based violence). It also shows that some vulnerabilities, such as migrant or indigenous status, are not commonly listed as the primary determinant of vulnerability but rather as one of multiple dimensions of vulnerability.

Importantly, many vulnerabilities interact with each other in compounding and intersecting ways. For example, lack of credit facilities, inadequate climate change information and adaptation knowledge, access to water for farming and livestock, inadequate government support for smallholder farmers, lack of property rights of female smallholders, and lack of access to markets all affected smallholder farmers' adaptation responses to combined mean precipitation decrease and drought in the Upper East Region of Ghana.^77^ In coastal Odisha, India, out-migration is a common response to compounding hazards (tropical cyclones and extreme precipitation), but gendered vulnerabilities intersect with caste, income, geographic location, age, and household membership to create heterogeneous responses that often exacerbate exclusion of women.^104^

The importance of how multiple non-climate stressors affecting vulnerabilities interact with compound climate events is also highlighted in more recent studies of human responses to super cyclones. Cyclone Amphan hit the Bay of Bengal in May 2020, when COVID-19 was at its peak in India and Bangladesh.^110^ This made conventional cyclone responses such as standard evacuation processes extremely difficult due to pandemic protocols of social distancing. Understanding these challenges of responding to compound climate events will be increasingly important for anticipatory action as there is a projected increased population exposure to Amphan-scale cyclones under future climates.^111^^,^^112^

In Uganda, farmers with more land, education, access to governmental extension, a non-farm livelihood, larger households, and older age had more capacity to buffer shock from drought and increased precipitation variability through increased assets and entitlements than poorer farmers who were more likely to engage in opportunistic behavior like casual laboring.^90^ Access to financial services and social capital has helped ethnic minority groups in upland northern Vietnam to manage when harvests have been compromised by extreme cold or drought conditions.^113^ Across Cameroon, Equatorial Guinea, and Rwanda, adaptation to the compound effects of extreme heat, agricultural drought, and increases in crop pests is influenced by the availability and access to forests and forest resources and the degree to which their livelihood strategies have diversified away from forest dependence.^114^

Interactions between vulnerabilities are generally described in the adaptation literature qualitatively (68% of articles). Spatially compounding vulnerabilities are described in the interactions between food insecurity, low income, and dwelling in high-risk areas for coastal communities in Tanzania affected by impacts of sea level rise and coastal flooding.^99^ Temporally compounding vulnerabilities are described in the interactions over time among food insecurity, immobility, chronic health challenges, and migrant status for communities affected by extreme heat in Bukavu in DR Congo^115^ and heat and drought in the Philippines.^74^

Inequality and injustice are often underlying patterns behind differential vulnerabilities, such as the intersection of multiple aspects of identity altering the way people experience discrimination or how historical injustices such as colonialism, racism, and sexism have shaped current exposure patterns and access to adaptation resources.^18^^,^^116^^,^^117^^,^^118^^,^^119^ These justice elements are often implied in the adaptation literature rather than being named explicitly, which reduces the potential for the field to recognize and resolve continuing inequities. Recent research has also unfolded how response risks span both adaptation and mitigation, for example the ways in which keeping households cool in conditions of extreme heat through use of air conditioning involves behavioral, financial, well-being, and health trade-offs with the potential for poverty traps for lowest-income individuals.^120^

Although it is common for articles to list more than one vulnerability that is relevant to the response to compound climate event, they rarely list all the relevant dimensions of vulnerability or the differential effects they have on other vulnerabilities or on responses. It is also common for indicators of vulnerability and exposure to be conflated across qualitative and quantitative studies reducing the accuracy and specificity of information for risk assessment. This information would be valuable to understand the constraints that contextualize the potential efficacy of response options across multiple contexts. This is particularly important given how frequently key dimensions of inequality (11 articles), governance (9 articles), government responsiveness (5 articles), informality policy/practices (5 articles), and migration policy (5 articles) were identified to affect responses to compound climate events through their impact on the background conditions driving vulnerability.

**Figure 3:**
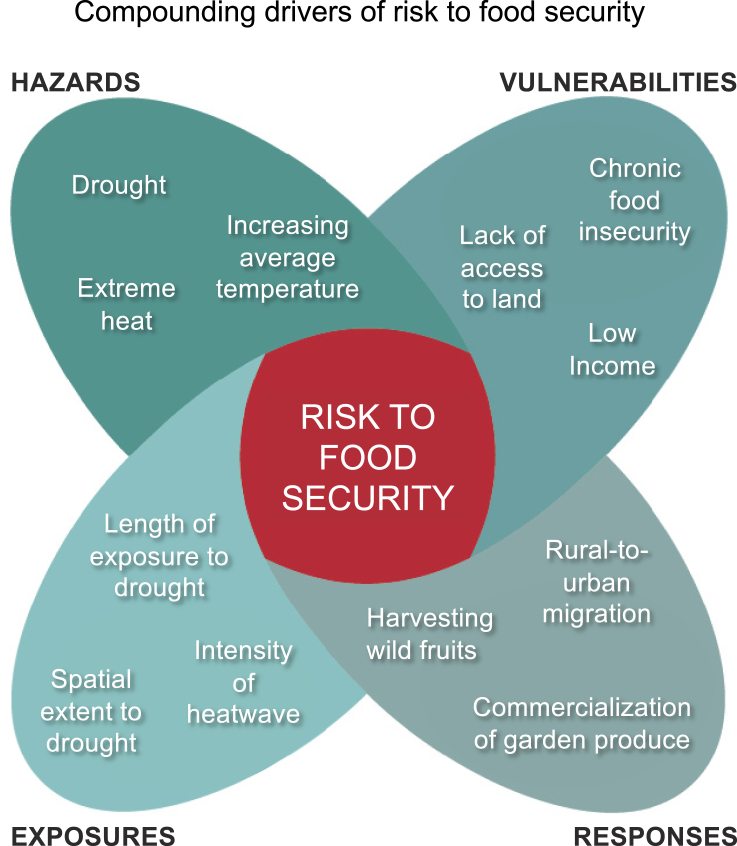
Compounding drivers of risk to food security highlighting the effect of multiple intersecting vulnerabilities Multi-variate drivers of risk within each of the four determinants of climate change risk documented in Masunungure and Shackleton.^72^

Figure 3 is populated with information from Masunungure and Shackleton,^72^ one of the 53 articles reviewed and discussed here. In this case, compounded drought and extreme heat (hazards) were abnormally intense and protracted and covered a large area (exposures). The severity of impacts to food security was largely determined by their limited and reactive adaptation options (responses) and by chronic food insecurity and lack of access to land for low-income communities (vulnerabilities) in South Africa. This case highlights the importance of responses that address critical vulnerabilities for marginalized communities and make the greatest contribution to risk reduction among the four determinants of risk. Combinations of harvesting wild fruits, commercialization of garden produce, and social grants provided an important safety net that reduced vulnerability and the overall impact.^72^ However, poverty and inequality compounded each other to place soft limits to *in situ* adaptation.^72^ As a result, these customary responses were unable to anticipate or fully reduce risk equitably. For many community members unable to cope, their only response was to leave the land and migrate to cities.

### Responses

**Figure 4:**
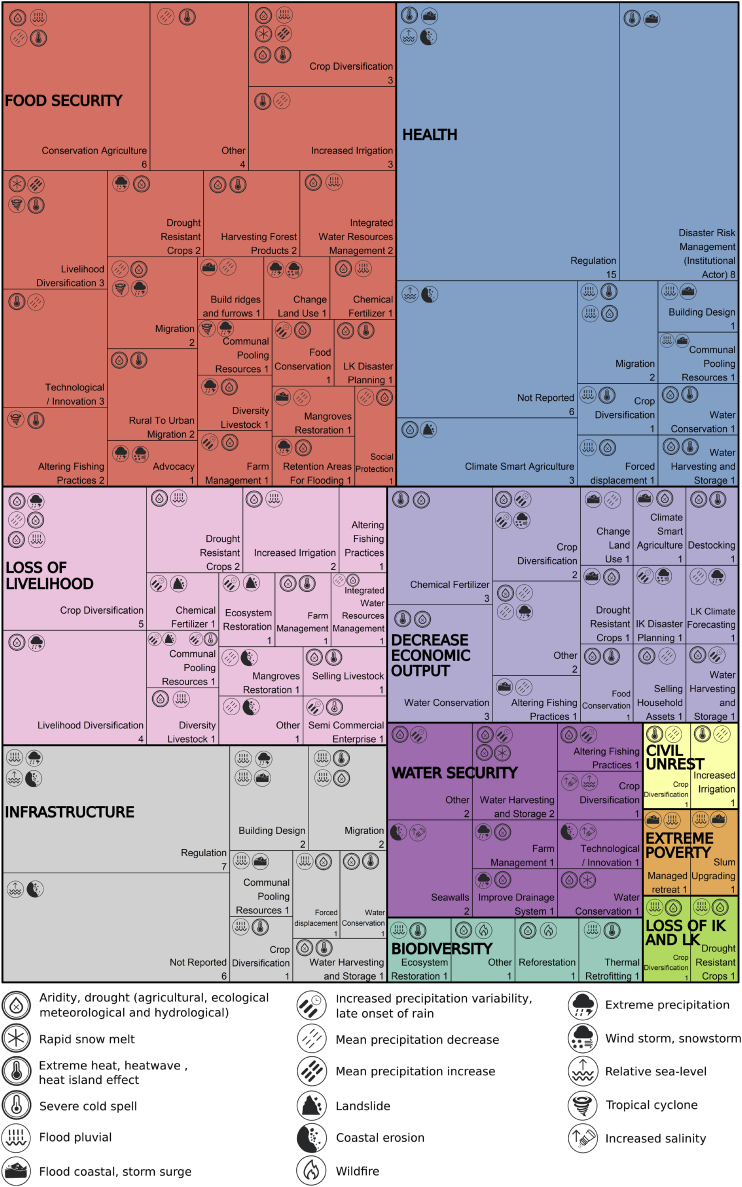
Identified responses to compound climate impacts and events and their relationship to Key Risks For the 10 most commonly identified Key Risks in the adaptation literature, all 45 classes of 175 adaptation-related responses are identified together with the compounding hazards to which they are responding. 17 hazards are shown by icons in the top left of each box and responses together with their article number count in the bottom right. Each set of two main compounding hazards is listed separately; for instance, for the Risk to Food Security, Conservation Agriculture responses targeted two sets of compounding hazards: (1) Drought and Flood pluvial and (2) Mean precipitation decrease and Extreme heat, heat wave, and heat island effect. Acronyms: IK - Indigenous Knowledge; LK - Local Knowledge.

There are at least 45 types of adaptation response to compound climate events documented in the adaptation literature (175 total documented responses in 53 articles) (Figure 4). The seven most common responses to compound climate impacts are all related to food security and include crop diversification,^77^^,^^88^^,^^121^^,^^122^ planting drought-resistant crops,^81^^,^^90^^,^^101^ altering fishing practices,^84^^,^^87^^,^^103^ regulation,^96^^,^^109^^,^^123^ livelihood or labor diversification,^121^^,^^122^ and water harvesting techniques/storage capacity.^124^^,^^125^

When linking the observed impacts documented in the articles to the IPCC AR6 Key Risks, compound climate events in the adaptation literature most commonly present severe risk to food security,^74^ risk to health,^71^ loss of livelihoods,^79^ and decreased economic output.^101^ In many cases Key Risks compound, for example, risk to food security commonly compounds with risk to livelihoods across smallholder farming communities.^76^^,^^80^^,^^126^ Across most of these Key Risks, the effect of compound climate events on water is a common driver of risk and a key focus of the adaptation response. For example, improved farm management practices translate to using less water-demanding agriculture in response to compounding agricultural drought and extreme heat in the Melamchi Valley of Nepal.^73^

There is increasing evidence of adaptation constraints, including both soft (68%) and hard (18%) limits.^9^^,^^39^ Soft limits reflect current constraints, where adaptation options are currently not available but may become available in the future, while hard limits are those where no further adaptation is possible.^39^ In the adaptation literature, 92% of the articles indicate limits of adaptation being reached. The broader GAMI dataset that generally considers responses to individual climate hazards indicated that 82% of articles included evidence that limits of adaptation have been reached. This 10% difference is indicative of the severity of risk associated with compound climate events and greater challenges of adapting to them.^86^^,^^97^^,^^127^

Examples of soft limits to compound climate hazards include lack of access to finance,^83^^,^^90^ unaffordability of new technologies,^90^^,^^99^ poor governance and government responsiveness,^122^^,^^127^ limited social or cultural capital,^71^^,^^73^^,^^113^ poverty and inequality,^72^ limited formal education,^79^ and loss of indigenous knowledge (IK) or local knowledge (LK).^9^^,^^98^

Examples of adaptation reaching hard limits include dislocation and relocation of households due to combinations of riverbank erosion and tropical cyclones^103^ and water conservation measures by smallholder farmers in rain-fed systems under compound drought and extreme heat.^75^ Among cases where hard limits had been reached, in 17% of studies multiple other soft limits were also documented to have been reached. This highlights the complexity of limits to adaptation to compound and cascading events and the importance of integrating responses in understanding risk. While some soft limits might be manageable with greater investment, concurrent hard limits can potentially undermine those efforts. Nevertheless, identifying limits can better inform the design and expectations of current and future adaptation options, particularly when efforts address the soft limits that are commonly associated with social dimensions of vulnerability.^9^

**Figure 5:**
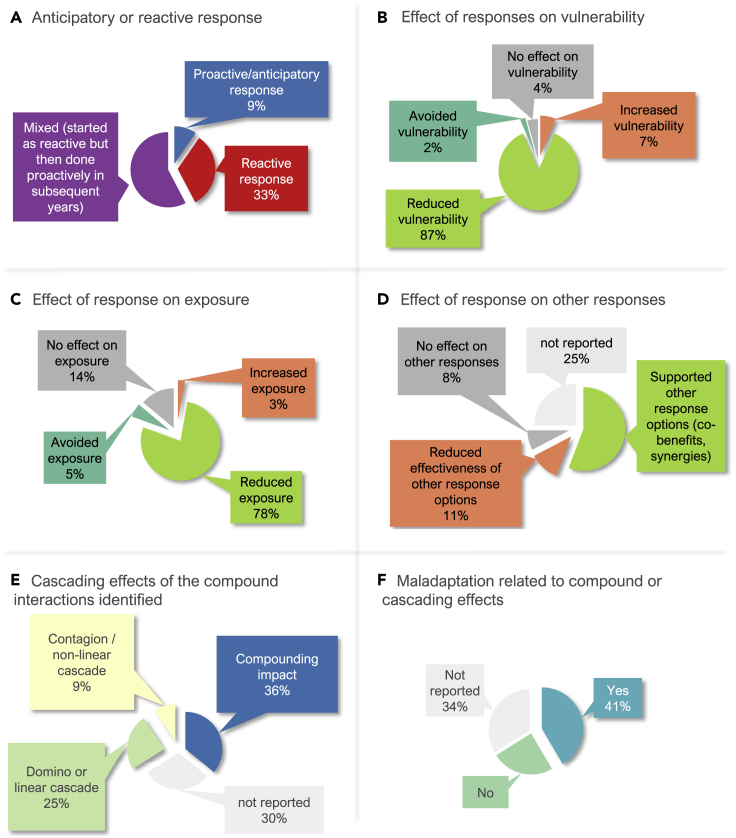
Effects and characteristics of responses to compound climate events (A–F) anticipatory or reactive responses; (B) effects of responses on vulnerability; (C) effects of responses on exposure; (D) effects of responses on other responses; (E) Cascading effects of the compound interactions identified; (F) Maladaptation related to compound or cascading effects (n = 175 responses).

Responses to compound climate events are generally reactive at first, but the literature shows that over time there is potential for responses to become more proactive (Figure 5A). For example, water harvesting and water-saving technology in response to the Cape Town drought was initially reactive^128^^,^^129^ but led to manifold responses across all levels of society which have improved the household and general water resilience of the city.^50^^,^^130^ Similar responses have been seen in adaptation to variable water supply resulting from successive combinations of drought and rapid snow melt in the Truckee-Carson River System, Western USA,^125^ in response to drought and extreme heat the Great Plains drought’s effect on McCook, Nebraska,^71^ and in response to flooding and wildfire in eastern Australia.^131^

Only 9% of articles describe anticipatory and proactive responses (Figure 5A). For example, harnessing ecosystem services to buffer communities against pluvial flooding, extreme heat, and coastal erosion, three Swedish coastal municipalities implemented and planned measures that support ecosystem-based adaptation that focused on enhancing biodiversity and ecological infrastructure.^127^ Proactive use of communal pooling resources, listening to the weather forecasts, and stockpiling food helped rural households in the Red River Delta of Vietnam respond to compounding flooding and storm surge.^86^^,^^97^

Eighty-seven percent of articles provide evidence of vulnerability reduction (Figure 5B). Changing planting dates and increased irrigation in response to extreme heat and mean precipitation decrease in the Indo-Gangetic Plains were noted to reduce vulnerability and exposure and supported other response options.^132^ One article demonstrated the importance of interactions between response and vulnerability determinants of risk through highlighting mapping of vulnerable locations as a proactive and anticipatory response for identification of cooling areas and public information on how to stay safe during heat waves.^133^

Most articles demonstrated how responses reduced (78%) or avoided exposure (5%) (Figure 5C). For example, in response to compound increased salinity and relative sea level rise in the Mekong Delta, risk of exposure to salinity intrusion was reduced through a combination of construction of seawalls, river dykes, sluice gates, and irrigation infrastructure.^97^

However, 7% of articles exhibit evidence that the response to compound climate events increased vulnerability. For example, in Bukavu, DR Congo, increased irrigation in response to extreme heat and decreased precipitation led to increased evapotranspiration losses with marginal benefits to productivity. The climate hazards, combined with a lack of alternative livelihoods, led to livelihood loss and a deepening poverty cycle.^97^^,^^115^

The complex relationship between hazards, exposures, social vulnerabilities, and responses is shown in Isle de Jean Charles, Louisiana, USA. This island is being inundated by sea level rise, hurricanes, coastal erosion canalization of the wetlands by oil and gas exploration, and the levees starving the Mississippi delta of sediment and through delta subsidence.^134^ A federal decision not to enclose the island within a levee left the islanders with few adaptation options, while another decision to not recognize the residents as a Native American tribe reduced their political power to self-govern.^135^^,^^136^ Relocation, either autonomously as households or as a coordinated community resettlement, has complex impacts on community members, as an adaptation loss and damage are created for different stakeholders simultaneously, such as coping with the loss of place attachment and emotional stress.^137^^,^^138^

How responses affect other responses is important for informing better adaptation to climate change and avoiding negative trade-offs in response options. Eight percent of articles indicated responses had no effect on other responses, while 25% of articles did not report on how responses affected each other (Figure 5D). Over half of the article showed that responses supported other response options, indicating positive synergies and co-benefits. For example, in response to compounding increased precipitation variability and windstorms in a sampaguita flower-growing community in the Philippines, integration of indigenous and scientific knowledge was a crucial process in livelihood disaster risk reduction and resilience building.^95^ In this case, resilience processes around food sufficiency and crop diversification empowered the marginalized community to escape chronic poverty and collectively act on other constraints including climate change-related risks.^95^ Across the 53 studies, 34% showed evidence of use of IK and LK in implementation of responses to compound climate extremes. This is higher than what was found in the full GAMI dataset where 24% of human adaptation-related responses to climate change included evidence of use of IK and LK.

However, 11% of articles also identified how selected responses reduced the effectiveness of other response options (Figure 5), highlighting how responses compound with one another. For example, in Jakarta, flooding results from multiple factors, one of which is climate change. In response to coastal and pluvial flooding compounded by sea level rise, flood policies in Jakarta are failing to mitigate risk for the city’s poorest populations and are instead compounding the risks they face from eviction and increased poverty.^102^ Impacts from compound climate hazards are increasing^62^ and are placing unprecedented constraints on response capabilities needed to target one hazard impact because resources are simultaneously needed to manage multiple other impacts and their synergistic or cascading effects.^139^^,^^140^ For example, the Cape Town drought presented a “shock within a shock” as reduced household water consumption in response to the drought compounded with reduced revenues collected by the municipality for water sales, placing severe constraints on additional finance needed for disaster risk management and the development of alternative water supply options.^29^^,^^50^ Such risks are further complicated by conflicting priorities across multiple responses considered and chosen by government and private entities. This creates multiple potential pathways to maladaptation and partial resilience.^130^^,^^141^

Of the documented adaptation responses, behavioral or cultural adaptation-related responses were the most common type reported (33%), followed by ecosystem-based or nature-based (25%), technological or infrastructural (25%), and institutional (17%). Articles frequently reported responses that involved multiple types of adaptation, for example, the installation of urban green roofs for cooling (nature-based and technological) or government-supported planting of drought-resistant seeds among subsistence farmers (behavioral and institutional). A typical behavioral response for smallholder farmers is the combination of crop diversification, diversification of income sources, and increased irrigation in response to extreme heat and mean precipitation decrease.^115^ Behavioral responses to compound climate events are observed on every continent and in small island states but most commonly in Asia (10 articles) and Africa (8 articles) in the adaptation literature. Behavioral responses, however, commonly display constraints on adaptation including lack of adequate technical knowledge and financial resources highlighting the importance of complementary and enabling institutional and technological responses.^78^ Ecosystem- or nature-based responses are most commonly observed in Asia (15 articles) and Africa (11 articles) and in 66% of North American-focused articles. An example of an ecosystem-based response is the planting of mangroves to minimize coastal flooding.^99^ Institutional responses are the least common type. Across Bangladesh, Bhutan, India, and Nepal, institutional responses include the development of several major policies and plans that directly or indirectly support other adaptation responses including using improved crop varieties, changing cropping patterns and planting seasons, and water conservation techniques.^78^ Climate-smart agriculture is an example of technological response employed together with a change from subsistence to semicommercial farm enterprises and changing water use modes in response to hydrological drought and increasing average temperature in Peru, Nepal, and India.^142^ Technological responses are most commonly recorded in the adaptation literature on Asia (38% of all technologically based responses).

These categories of adaptation-related responses to compound climate impacts matter because behavioral, ecosystem-based or nature-based, technological or infrastructural, and institutional types of responses have varying effectiveness and feasibility across different contexts. For example, at current global warming levels, the IPCC 6th Assessment assessed human migration in Africa to have high potential effectiveness as a behavioural response but low feasibility due to economic, institutional, and technological barriers.^10^^,^^18^ Furthermore, the effectiveness of migration in response to climate change displayed across the entire GAMI dataset for Africa is higher for West and Eastern Africa but low for southern and central Africa.^10^^,^^18^ But a recent study has shown temporally compounding droughts exacerbate structural vulnerabilities and limit migrants’ adaptation options, including long-range climate mobility by agricultural communities.^143^

A similarly differentiated pattern emerges for technological responses, like bulk water infrastructure development and financial investment in agriculture. For both of these, potential effectiveness is high, while feasibility is low due to institutional and technological dimensions constraining adaptation.^10^^,^^18^ Furthermore, broader literature has shown that some technological approaches can pay little attention to equity and justice, as in the case of the dominant technological approach to managed retreat.^144^ These findings highlight the importance of understanding how context, including system capital and connectedness,^58^ affects differential effectiveness and feasibility of responses to compound climate events.

### Cascading effects and maladaptation

The adaptation literature identifies a range of cascading effects between determinants of climate change risk under conditions of compound climate events (Figures 5E and 5F). The most commonly described in the literature is the domino, or knock-on, effect where one determinant of risk affects another, which in turn affects one more in a relatively linear manner. For example, domino social effects are displayed in shifting the location of houses from the upland to lowland areas in order to avoid the hardship of daily water management^124^ and reduced contribution of subsistence farming to support food security as a result from livelihood diversification, and in turn, increased dependency on imported food.^99^

Two other cascade effects are identified. Over one-third of articles describe a type of compounding interaction, where two or more determinants of risk act together to severely affect one determinant (Figure 5E). For example, driven by the effects of compound extreme precipitation and snowstorm, together with social-institutional change (i.e., privatization of rangeland use rights, sedentarization, pasture degradation, and fencing), rangeland degradation and severe snowstorms negatively affected livestock production, and tens of thousands of herders in the Tibetan pastoral systems suffered heavy losses of livestock and herders’ livelihoods.^93^ New research has also advanced understanding of the ways in which spatially cascading impacts of “new-normal” high tide flooding interact with transportation-route options and decisions, in turn modulating access to livelihoods and essential services.^111^

Two articles^71^^,^^100^ describe a type of contagion cascade where the effects of the interaction between one or more determinants proliferate to affect multiple determinants of risk (Figure 5E). For example, impacts from compound extreme heat and drought in McCook, Nebraska, had ripple effects onto others reducing lake and pond levels and overall surface water supply, increased reliance on groundwater, and diminished fish and wildlife habitat.^71^ In this case, irrigators experienced a shortage early on leading to highly priced and increasingly scarce hay, forcing ranchers to relocate, cull, or sell their herds at below-normal prices. Meanwhile, recreational visitors to neighboring lakes were deterred by reduced water levels and toxic blue-green algae, outdoor activities declined, and fishing suffered. Producers noted that high land values, singly and in combination with severe drought conditions, affect the ability of small landowners to remain operational, which has cascading effects on various agribusinesses. Resulting market fluctuations included a decrease in market price of hay, corn, soybeans, and cattle.^71^

The Black Summer fires of 2019–2020 in eastern and southern Australia demonstrate the far-reaching cascading impacts across natural and human domains that were exacerbated by resource constraints in response capacity which have been identified as a barrier to containing fires effectively.^131^ A positive result of the inquiry into the Black Summer fires has been new institutions to redress the response, planning, and information management shortfalls and to implement a national approach to national disasters. Such adaptations and enablers to manage cascading risks include land management, communications, understanding interactions (between fire, fuel, weather, human behaviors, and infrastructure), and institutional options that improve response enablers including governance, accountability, and coordination between agencies.^145^

Maladaptation is observed in 41% of articles (Figure 5F). For example, the compounding effects of tropical cyclones and extreme heat had a negative impact on fishers of the Padma River.^103^ However, embankments constructed to control riverbank erosion disconnected the Padma River from the natural depression and associated floodplains, interrupting refugia for small size fish, eggs, larvae, and juveniles. As a result, fish production was reduced by 20%.^103^ In a recent review, AghaKouchak et al.^27^ highlight that planned actions in response to more frequent extremes often involve construction of new developments such as building larger dams, constructing seawalls, or increased energy generation for cooling purposes. However, they show that these responses have trade-offs with climate change mitigation and often lead to even more emissions and have negative impacts on local-regional extreme events.^27^ This work emphasizes the complexity of responding to compound climate events. As our knowledge of such events increases, we may better avoid maladaptation.

### Limitations of the study

The 53 articles reviewed here focused on compound climate hazards and are a very small fraction of the vast literature on adaptation to climate change impacts and to climate risk more broadly.^8^ This review shows that there is an emerging understanding across the global adaptation literature of the severity of compound climate events and the challenges of adaptation. However, conceptual and empirical gaps need to be urgently filled so that more effective adaptation can be achieved that links human responses to exposures, hazards, and vulnerabilities.

This article has focused on human responses to compound climate impacts. Where the literature has identified a role of ecosystem-based adaptation or nature-based solutions has been highlighted. However, responses to compound climate impacts are necessary for all of life on earth including ecosystems^146^^,^^147^^,^^148^ and the ecological infrastructures they provide.^149^^,^^150^ These broader response domains also need to be better understood for how they relate to human adaptation to climate change. For example, healthy ecosystem functioning mediates human adaptation, but ecosystems are themselves also directly affected both by the compound climate impacts^151^^,^^152^ and by human responses to them.^153^^,^^154^ Examples from this review highlight that human responses depend on healthy and functioning ecosystems.^86^^,^^97^^,^^107^^,^^127^ Deeper understanding of how such interactions affect climate risk and adaptation therefore merits further research.

### Conclusion

The greatest risks from a changing climate may not come from individual climate impacts and risks but from the interactions and interdependencies between cascading and compound climate impacts and socio-economic and geopolitical vulnerabilities—in other words, the nexus between compound climate hazards, exposures, existing intersectional vulnerabilities, and multiple types of human responses. These interactions can be mapped via available tools and analytic methods such as the Circle tool for infrastructure and system interactions,^139^^,^^140^^,^^155^ exploratory modeling analysis with integrated models that capture interactions between sectors,^58^ pathways analysis,^36^ and predictive modeling through the use of artificial intelligence techniques.^20^ However, compounding risk increases complexity, and existing tools to support decision makers would need to be further tailored to systematically assess interactions of risk and responses across space and time, to support the development of adaptation plans that properly account for compounding risks. This will not, however, rule out surprises, which points to the need for warning and response capability in the context of learned experience. Analogues exist from which we can learn. Disruptions in global food supply chains arising from a combination of COVID-19 and the war in Ukraine are demonstrations of the types of risks we can expect from climate change and a reminder of the importance of compound and interacting risks.

Kemp et al.^156^ highlight the potential for catastrophic and extreme climate scenarios leading to societal collapse, with compound events and their interactions as key drivers of social fragility. Consistent with Kemp et al.,^156^ we contend that catastrophic climate scenarios are not entirely unpredictable and argue that there is significant potential to identify the common drivers and mechanisms underpinning diverse compound and catastrophic risk scenarios from other domains of learning. In particular, we highlight the importance of integrating consideration of compound and cascading risks into adaptation planning. In doing so, and consistent with the recent IPCC Sixth Assessment Report, we situate adaptation responses as a core component of risk alongside climate hazards and human exposure and vulnerability. These findings highlight that averting catastrophic climate scenarios is a function of climate change adaptation and actions addressing existing human vulnerability. Importantly, however, the findings also show how the efficacy of adaptation is influenced by the ways in which we choose to adapt, including choices to delay or avoid responses to climate change.

These findings highlight the importance of understanding how context, including system capital and connectedness, affects differential effectiveness and feasibility of responses to compound climate events. Addressing many of the factors affecting the soft limits to adaptation can provide an entry point for more effective responses to compound extreme events—for example, improving access to finance, affordability of new technologies, and good governance and government responsiveness; enhancing social, gender, and cultural capital; reducing poverty and inequality; and boosting climate change literacy and education and safeguarding indigenous and traditional knowledge, while better addressing inequality and other economic vulnerabilities to improve climate change responses.^157^ This requires greater attention to the complexity of interactions affecting risk and application of both qualitative and quantitative studies to empirically demonstrate these effects across different scales and contexts. Failure to do so will underestimate the urgency and severity of risk from responses to climate change, fail to ensure safeguards for vulnerability and exposure concerning responses (both for adaptation and mitigation), and miss the opportunity to better inform how responses can effectively reduce risk that can unlock inaction, policy inertia, and uninformed adaptation and mitigation responses to climate change and enable climate resilient development.

## STAR★Methods

### Key resources table

**Table:** 

REAGENT or RESOURCE	SOURCE	IDENTIFIER
**Deposited data**		

All articles identified through the GAMI process and coded for this analysis	Mendeley Data	https://doi.org/10.17632/yxdw47555s.2

### Resource availability

#### Lead contact

Further information and requests for resources and reagents should be directed to and will be fulfilled by the lead contact, Nicholas P. Simpson (nick.simpson@uct.ac.za).

#### Materials availability

Full list of all articles identified through the GAMI process and coded for this analysis have been deposited at Mendeley Data and are publicly available as of the date of publication from Mendeley Data, V2, https://doi.org/10.17632/yxdw47555s.2.

### Method details

Using combined human and machine learning approaches, we conducted a global systematic stocktake on human adaptation to climate change (2013-2019).^38^ A search of Scopus, Web of Science, and Google Scholar based on search terms that combined concepts of climate change and adaptation or adaptation-related responses identified nearly 50,000 articles published between 2013 and 2019.^158^ After preliminary screening, 1,682 articles were included in the final Global Adaptation Mapping Initiative (GAMI) dataset. Each article was then coded by at least two coders by a team of 120 researchers.^159^ The GAMI dataset has led to a broad range of research outputs, including a systematic global stocktake of evidence on human adaptation to climate change,^8^ assessment of feasibility and effectiveness of climate adaptation options across Africa,^10^ assessment of the responses to climate-related water scarcity in Africa,^12^ trade-offs and synergies across global climate change adaptations in the Food-Energy-Water nexus,^160^ assessment of the role of Indigenous Knowledge and Local Knowledge in water sector adaptation to climate change in Africa,^161^ adaptation in conflict-affected areas,^162^ a global assessment of policy tools to support climate adaptation,^163^ adaptation gaps in mountain regions,^164^ equity in human adaptation-related responses,^165^ review of the evidence of constraints and limits to human adaptation,^39^ the effects on public health of climate change adaptation responses,^11^ and review of climate change adaptation to extreme heat.^34^ Yet none of the analysis of GAMI to date has explored responses to compound climate events nor how responses compound with each other to affect risk.

The team set out to identify compound and cascading hazards and response interactions affecting climate change risk in the scientific climate change adaptation literature published between 2013 and 2019. GAMI had already coded all individual hazards and adaptation-related responses documented in the articles. However, the full GAMI dataset needed to be screened to find which articles documented responses to compound climate events or their impacts and for specific analysis concerning responses to compound climate impacts. We therefore screened 1682 GAMI articles using NVivo key word search (compound∗ OR cascad∗) and selected the sub-set of human adaptation papers that displayed responses to compound climate events. This initial step identified 113 articles for potential full text coding for compound effects. A team of 11 coders then explored this set of papers with a full text readthrough of each article for responses to compound climate events, and how responses compound with each other to affect risk. After this second round of screening, a further 60 articles were excluded based on their lack or insufficient documentation of responses to one or more compound climate events. This left 53 articles for full analysis.

Each article was coded through a blinded process by two different coders (see Table S1). Codes were then adjudicated and reconciled by a third coder where differences were noted. The review identified compound hazards and the nature of the interaction between them (e.g., spatial, temporal). The most relevant climate-related precondition to the compound interaction was also identified (e.g., false-spring). The two most significant responses to the compound climate events were then identified (the most prominent and effective responses), noting the effectiveness of the interaction of the response with the hazard(s) of the compound event. These responses were also considered for their risk-related effect on hazards, responses, exposures and vulnerabilities (e.g., supported other response options; reduced effectiveness of other response options; no effect on other responses). Notable response preconditions were identified (e.g., government responsiveness or co-occurrence of non-climate hazard such as earthquake), together with dimensions of vulnerability and exposure affecting responses (e.g., income; migrant status); and preconditions affecting vulnerability or exposure (e.g., conflict/insecurity; migration policy). How these dimensions of vulnerability and exposure affect each other and vary across populations makes some populations more vulnerable or exposed than others. Exposure is about the opportunity to be impacted while vulnerability is about the characteristics of what is impacted. Articles where then interrogated for cascading or compounding effects arising from the compound hazard and response interactions identified including domino or linear cascade (1 -> 2 -> 3 -> 4 etc.); contagion or non-linear cascade (1 -> 3 -> 6- > etc.); and compounding impact (3 -> 1). Reflecting on the outcomes of responses, analysis explored evidence of maladaptation as a direct consequence of the responses identified.

In order to locate empirical responses within a broader understanding of risk, the observed impacts in each paper were categorized by the coding team according to the ‘key risks’ identified by the IPCC sixth Assessment, such as risk to biodiversity or food security.^9^ A Key Risk is defined by the IPCC as a potentially severe risk because of dangerous interference in the current climate system.^9^ Key Risks will likely become more severe with increased warming due to associated changes in the nature of hazards and/or of the exposure or vulnerability of societies or ecosystems to those hazards. They also may become severe due to the adverse consequences of adaptation or mitigation responses to the risk.^9^ Although articles did not generally use the IPCC’s Key Risks terminology, Key Risks were used in the analysis of each article to better understand why people were responding beyond simply how they were responding. This is based on the premise that human responses to compound climate events are not random but can be deliberate investments in avoiding or reducing risk to things they value. Key Risks also provide a useful range of categories for a review level assessment of the literature as they enable reflection of which sectors are currently documenting the most responses to compound climate impacts.

Recognising that the adaptation literature may not include the breadth of illustrations of compound climate events, nor responses to them necessary to enhance our understanding of adaptation to climate change, we also include 11 extended examples in the discussion of the results through reference snowballing from the underlying literature of the recent IPCC sixth assessment Working Group 2 report.^35^ These examples reflect the authors’ contributions to and insights drawn from the underlying literature they assessed across the Summary for Policy Makers, Technical Summary, Point of departure and key concepts (Ch. 1), Poverty, livelihoods and sustainable development (Ch. 8), Africa (Ch. 9), Asia (Ch. 10), Australasia (Ch. 13), Europe (Ch. 13), Key risks across sectors and regions (Ch. 16), Decision-making options for managing risk (Ch. 17), and Synthesis Report of IPCC sixth assessment.

## Data Availability

• This paper does not report original code. • This paper does not report original code. Coded data for this analysis have been deposited at Mendeley Data and are publicly available as of the date of publication. Accession numbers are listed in the key resources table. • Any additional information required to reanalyze the data reported in this paper is available from the lead contact upon request.

## Supplemental information

Table S1. Coding instructions for analysis of GAMI data
